# Comparison of Left Ventricular Global Strain in Anterior and Non-anterior Wall Myocardial Infarction With CMR Tissue Tracking

**DOI:** 10.3389/fphys.2020.530108

**Published:** 2020-12-10

**Authors:** Shuhao Li, Lei Zhao, Aijia Lu, Jie Tian, Lianggeng Gong, Xiaohai Ma

**Affiliations:** ^1^Department of Radiology, Beijing Anzhen Hospital, Capital Medical University, Beijing, China; ^2^Department of Medical Imaging Center, The Second Affiliated Hospital of Nanchang University, Nanchang, China; ^3^Department of Interventional Therapy, Beijing Anzhen Hospital, Capital Medical University, Beijing, China

**Keywords:** cardiovascular magnetic resonance, myocardium infarction, anterior wall ventricle, left ventricular, tissue tracking

## Abstract

Left ventricular (LV) myocardial dysfunction occurs after myocardial infarction (MI) is associated with the location, infarct size, and transmurality degrees of MI. The myocardial strain is a sensitive index used for the quantification of myocardium dysfunction. This study used Tissue-Tracking to evaluate whether the different location of MI would result in different myocardial dysfunction. One hundred patients diagnosed with MI who underwent cardiovascular magnetic resonance examination were included. The tissue-tracking indices, LV global radial strain (GRS), global circumferential strain (GCS), global longitudinal strain (GLS), and the infarct size (IS,% of LV mass) were quantified. There were 42 cases of anterior wall MI (AWMI) and 58 cases of non-anterior wall MI (NAWMI). The GCS of AWMI was significantly lower than that of NAWMI (*P* = 0.036). In the same level of infarct size, the myocardial strain of AWMI was not significantly different from NAWMI group (*P* > 0.05). The GRS and GCS were significantly different between transmurality > 50% group with transmurality ≤ 50% group (*P* < 0.05). The present study demonstrated that LV MI is associated with reduced myocardial strain, and the infarct size and degrees of transmurality were both related to the decline of myocardial strain in patients with MI.

## Introduction

According to the 2013 ACCF/AHA guideline for the management of ST-elevation myocardial infarction, anterior wall myocardial infarction (AWMI) is a risk assessment index of Major Adverse Cardiovascular Events (MACE) (O’Gara et al., 2013). MI most likely occurs in the domain of the left anterior descending artery (LAD) (Park et al., 2014; Postma et al., 2015; Stone et al., 2016), which supplies nearly two-thirds of the whole heart blood (Bastiany et al., 2017). Infarct size is the percentage of infarcted myocardial mass to LV total mass. Transmurality is the degrees of sub-endocardium enhancement signal extending to the epicardium (Ogawa et al., 2017). Previous studies have suggested that the prognosis of patients with AWMI is significantly worse compared to patients with inferior wall MI (Geltman et al., 1979; Thanavaro et al., 1982; Hands et al., 1986; Bourke et al., 1988; Stone et al., 1988). However, other research has found no association between patient prognosis and infarct size (IS) or transmurality (Geltman et al., 1979; Thanavaro et al., 1982; Hands et al., 1986; Bourke et al., 1988; Stone et al., 1988).

Many methods have been developed to evaluate the myocardial function after MI. Left ventricular eject fraction (LVEF) is a well-accepted index for calibrating heart function assessment. However, LVEF is an index which reflects the macroscopic changes of the left ventricle, the subtle and regional myocardial changes during disease progression cannot be examined by LVEF. The myocardial strain is a new index for assessing the LV function, it revealed the subtle pathological changes of myocardium sensitively. The myocardial strain is the change in length of an object within a specific direction relative to its initial (often end-diastolic) length (Claus et al., 2015). The endocardium and epicardium outline were traced manually at the end-diastolic phase as the region of interest, marks each voxel in the region of interest, records its trajectory, and calculates the changes of each segment or the whole myocardial motion. The strain was divided into radial, longitudinal, and circumferential directions, representing the displacement in these three directions. CMR Tissue-Tracking (TT) was used to do the strain analysis and describe myocardial deformation in systole and diastole. The present study aimed to test whether different locations of MI would result in different myocardial dysfunction using Tissue-Tracking.

## Materials and Methods

### Participant Population

The study was approved by the Beijing Anzhen Hospital ethical committee, and written informed consents were obtained from all patients. The investigation conformed with the principles outlined in the Declaration of Helsinki. A total of 100 patients diagnosed with MI who underwent CMR examination between September 2015 and September 2017 were included. The inclusion criteria were the following: (1) coronary angiography showed a definite presence of coronary obstruction; (2) increased cardiac biomarkers (preferably troponin) in the acute period, at least one value exceeded the upper limit of the reference value or at least one evidence of myocardial ischemia; (3) coronary CTA showed the definite presence of coronary occlusion; (4) CMR perfusion and late gadolinium-enhanced (LGE) revealed myocardial fibrosis. The exclusion criteria were arrhythmia, poor breathing coordination, renal insufficiency, and contraindications for MRI. In the control group, healthy people with age and gender-matched with MI group, without a family history of cardiovascular disease, and abnormal founding in ECG and transthoracic ultrasound examination were selected.

**FIGURE 1:**
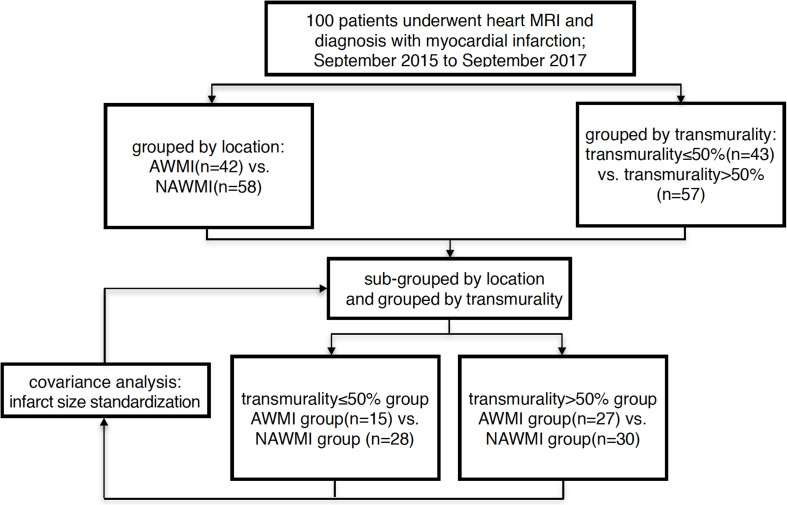
Flow chart of grouped method AWMI: anterior wall myocardial infarction. NAWI, non-anterior wall myocardial infarctions.

Patients with MI were grouped according to two factors: infarct location and the degrees of transmurality. Patients were divided into the AWMI group and the NAWMI group based on the infarct location; into transmurality ≤ 50% group and transmurality > 50% group based on the degrees of transmurality (Ogawa et al., 2017). 50 healthy volunteers of the same age and gender were recruited in the control group (Figure 1).

### CMR Acquisition

CMR scans were performed on a 3.0T MR scanner (Verio, Siemens, Germany) with 32 channel surface phased-array heart coil. A CMR sequence using the Steady-State Free Precession Sequence (SSFP) technique was used to obtain cine images at the short axis, two-chamber, three-chamber, and four-chamber views on LV under breath-holding. Imaging parameters included FOV 286 × 340 mm, matrix 216 × 256, and TR/TE 3.4/1.7 ms. A retrospective vectorcardiogram (VCG) was used as a trigger. A total of 25 phases were reconstructed in each cardiac cycle. In the short axis, 10–12 slices were collected for each participant.

### Image Analysis

**FIGURE 2:**
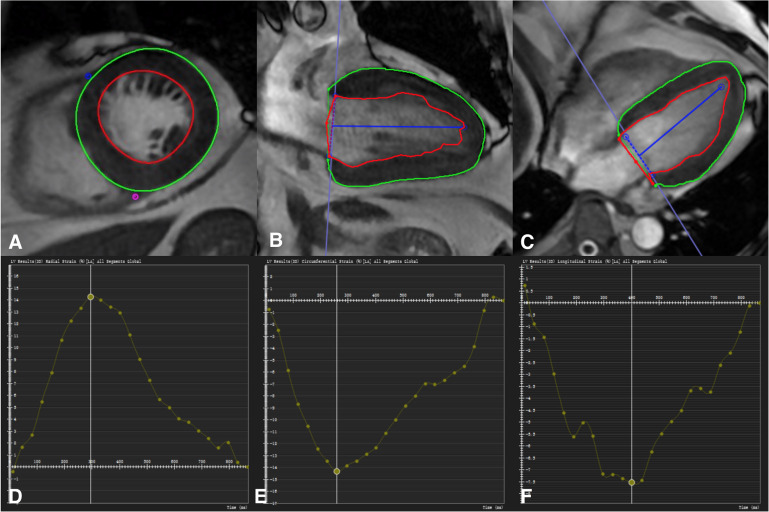
Manual delineation the border of endocardium and endocardium at the diastole image **(A–C)**. The marker was placed at the junction of left and right ventricle **(A)**. Defining the long axis of the ventricle **(B,C)**. Take the global peak radial strain **(D)**, circumferential strain **(E)**, and longitudinal strain **(F)** value for statistics.

All strain parameters were quantified by two experienced observers blinded to all patient data. These analyses were carried out retrospectively using CVI (cmr42 v5.6.4, Circle Cardiovascular Imaging Inc., Calgary, Alberta, Canada). Tissue-Tracking was used to analyze LV myocardial strain from short-axis, two-chamber, three-chamber, and four-chamber cine images. Briefly, LV endocardial and epicardial borders were manually traced in the multi-plane, excluding the aorta and LV outflow tract at the end-diastolic phase (Claus et al., 2015). The software automatically tracks on-screen pixels during the cardiac cycle. The peak value of radial strain, circumferential strain, and longitudinal strain was set as the GRS, GCS, and GLS for statistical analysis, respectively (Figure 2).

**FIGURE 3:**
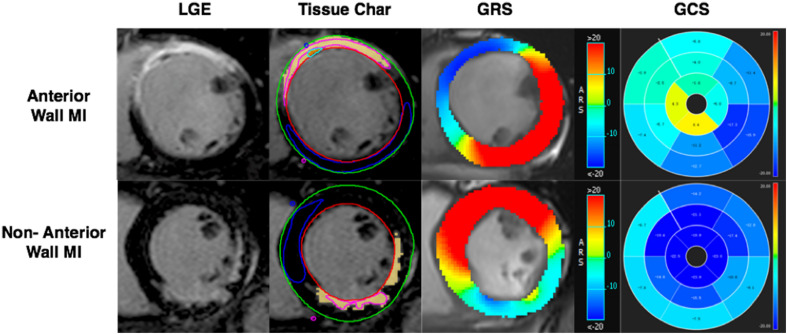
AWMI: CMR-LGE demonstrated MI involving anterior and partial septum wall. *Tissue Char* ROI view, the outline of endocardium and epicardium drew firstly, then the boundary point was set of the septum with the free wall. The pink counter was used to set in the hyperintensity area, the blue counter was set in the hypointensity area. The software will run the infarct size automatically. GRS of anterior MI was significantly decreased (blue part). 16 AHA segment map showed the GCS of anterior significantly decreased (green part). NAWMI: CMR-LGE demonstrated MI involving the inferior wall. Tissue Char ROI sketch view. GRS of non-anterior MI had significantly decreased (blue-green part). Sixteen AHA segment map showed the GCS of non-anterior had significantly decreased (nattier blue part). LGE, late gadolinium enhanced; GRS, global radial strain; GCS, global circumferential strain; GLS, global longitudinal strain.

LVEF was measured using the CVI short 3D module by drawing the endocardium and epicardium in the diastolic and systolic phases. Using the CVI Tissue char module calculated the infarct size by outlining infarct myocardium and normal myocardium (Figure 3). The infarct area was defined as five or more standard deviations (SD).

### Statistical Analysis

All analyses were performed using the SPSS (v23.0). Measurement data were shown as “mean ± SD.” *Levene* test was used for homoscedasticity. An independent sample *t*-test was used to compare the MI group to the control group, the comparison for location, and the transmural and infarct size. Covariance analysis was used for excluding the infarct size influence. *P* < 0.05 was considered statistically significant. The reproducibility of strain analysis was assessed using the Intraclass Correlation Coefficient (ICC) for a subset of 30 participants randomly selected by SPSS. The ICC of 0.75 or greater was considered excellent; 0.75–0.40 as moderate; and less than 0.40 as poor.

## Results

### Participant Characteristic

A total of 100 MI patients (92 males and 8 females, mean age 54 ± 12 years) and 50 healthy volunteers (46 males and 4 females, mean age 49 ± 12 years) were included in this study. There were 42 anterior MI patients and 58 non-anterior MI patients (10 inferior walls; 20 lateral walls; 8 septal walls; 20 multiple non-anterior walls) (Table 1). 59 patients were in the chronic stage (>3 months), and 41 patients were in the acute stage (≤3 months).

**TABLE 1:** Baseline characteristic of the study population.

	AWMI (*n* = 42)	NAWMI (*n* = 58)	Control (*n* = 50)
	Mean or count (SD)	Mean or count (SD)	Mean or count (SD)
**Male/Female**	39/3	53/5	46/4
Age (years)	53 (12)	54 (12)	49 (12)
Height (cm)	171 (6)	171 (6)	168 (6)
Weight (kg)	73 (11)	72 (10)	69 (10)
BMI (kg/m^2^)	25 (3)	24 (2)	24 (2)
LVEF (%)	45 (3)	49 (2)	63 (1)
**Treatment**			
Acute PCI	14	21	NA
Chronic PCI	4	6	NA
PTCA	NA	4	NA
CABG	6	1	NA
PAT	1	NA	NA
Medical	20	21	NA
**Comorbiditries**			
Hypertension	14	23	NA
Diabetes	9	12	NA
Hyperlipidemia	3	13	NA
Arrhythmia	4	7	NA
Liver dysfunction	2	2	NA
Hypothyroidism	1	NA	NA

### The Myocardial Strain Indices Comparison of AWMI, NAWMI, and Control Group

**FIGURE 4:**
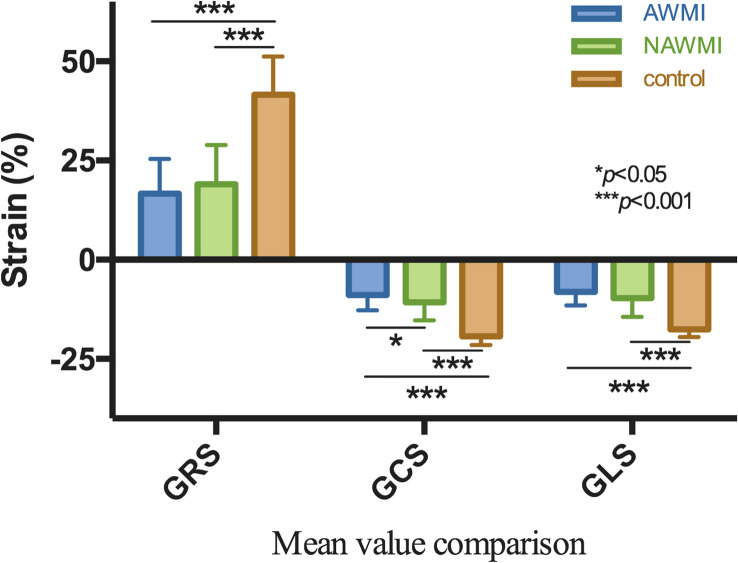
Comparison of GRS, GCS, GLS between AWMI and NAWMI, excluding the MI group were significantly different from the control group, the GCS was also significantly different between anterior with non-anterior MI. The mean GCS of AWMI was lower than NAWMI.

The GRS, GCS, and GLS of the MI group showed a remarkable decline compared with the control group (*P* < 0.001), indicating the patients’ LV function after MI was worse than the control group. The GRS, GCS, and GLS of the control group were compared with that of the AWMI group and the NAWMI group, both were statistically significant (*P* < 0.001). It means the LV function of the patients after MI were significantly decreased. Besides, the GCS of the AWMI group was significantly lower than that in the NAWMI group (−9 ± 10 vs. −11 ± 4, *P* = 0.036). It demonstrated that the LV function of the AWMI group was significantly decreased than the NAWMI group. Moreover, the infarct size of the AWMI group was larger than the NAWMI group (22 ± 9 vs. 14 ± 9, *P* < 0.001) (Table 2 and Figure 4).

**TABLE 2:** Comparison of AWMI, NAWMI, and control group (%).

	MI (*n* = 100)		Control (*n* = 50)	*P*-value		
	AWMI (*n* = 42)	NAWMI (*n* = 58)		AWMI vs. NAWMI	AWMI vs. control	NAWMI vs. control
GRS	17 ± 9	19 ± 10	42 ± 10	0.217	<0.001	<0.001
GCS	−9 ± 10	−11 ± 4	−20 ± 2	0.036	<0.001	<0.001
GLS	−8 ± 3	−10 ± 5	−18 ± 2	0.550	<0.001	<0.001
IS	22 ± 9	14 ± 9	–	<0.001	–	–

### Comparison of Myocardial Strain in Cases With Different Transmurality Degrees

**FIGURE 5:**
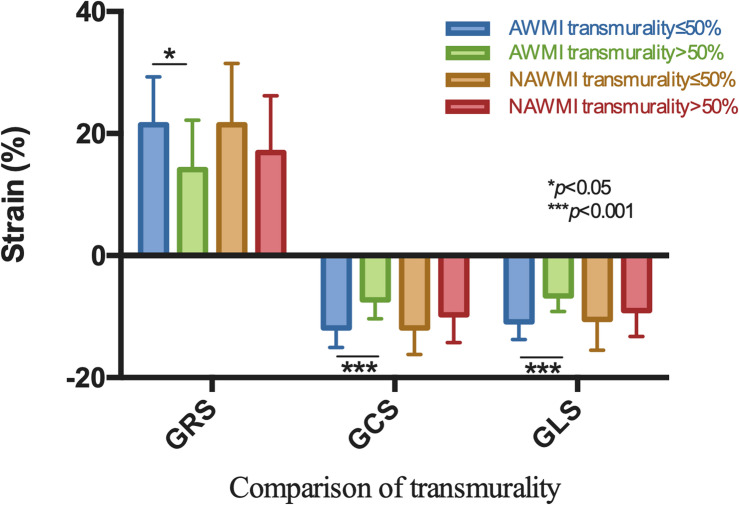
Comparison by the site of MI showed GRS, GCS, and GLS were significantly different with respect to different transmurality degree in AWMI.

A significant difference in the myocardial strain was observed between different transmurallity degrees (*P* < 0.05) (Table 3 and Figure 5). LV function of the transmurality > 50% group was worse than the transmurality ≤ 50% group.

**TABLE 3:** Comparison of transmurality degrees in all patients (%).

	Transmrality ≤ 50% (*n* = 43)	Transmrality > 50% (*n* = 57)	*t*	*P*-value
GRS	21 ± 9	16 ± 9	3.223	0.002
GCS	−12 ± 4	−9 ± 4	−4.058	<0.001
GLS	−11 ± 4	−9 ± 4	−3.348	0.001
IS	13 ± 8	17 ± 8	−4.551	<0.001

Patients were grouped by the transmurality degree and then sub-grouped by the location of MI. The GCS and GLS were significantly reduced in the AWMI group compared to the NAWMI group (*P* < 0.05) (Supplementary Table 1). Infarct size in the AWMI group was significantly larger than the NAWMI group, which both in the transmurality ≤ 50% group (17 ± 7 vs. 10 ± 8, *P* = 0.006) and the transmurality > 50% group (25 ± 9 vs. 17 ± 8, *P* = 0.001).

### Comparison of Standardization the Infarct Size

The present study used covariance analysis and used infarct size as a covariate. After standardization, there was no statistically significant difference in myocardial strain between different infarction locations (*P* > 0.05) (Supplementary Table 2).

Patients were grouped by the transmurality degree and then sub-grouped by the location of MI. There was no significant difference between myocardial infarctions with a different location (*P* > 0.05) (Supplementary Table 3).

However, the dysfunction of LV in the transmurality > 50% group was severe than the transmurality ≤ 50% group, which indicated by GRS (20 ± 1 vs. 16 ± 1, *P* = 0.041) and GCS (−11 ± 1 vs. −9 ± 1, *P* = 0.013) (Supplementary Table 4).

### Reproducibility

Thirty randomly selected patients were reanalyzed for the intra- and inter-observer feasibility and reproducibility. As shown in Supplementary Table 5, strain value and infarct size had an excellent agreement between intra- and inter-observer (ICC > 0.9).

## Discussion

The present study indicated that: (1) There is no relationship between post infarction myocardial strain reduction and infarction location; (2) Post-infarction strain reduction is related to transmurality or infarct size.

Our data demonstrated that the myocardial strain of patients who suffered from MI was remarkably reduced. Besides, the GCS of the AWMI group was significantly lower than that of the NAWMI group. Studies have shown that GCS reflects the degrees of transmurality MI (Mangion et al., 2017). Myocardial fibers were composed of three layers (circumferential, oblique, and longitudinal layer), which are arranged from the outside to the inside, i.e. the sub-epicardium is a circumferential fiber, the middle layer is an oblique fiber, and the sub-endocardium is a longitudinal fiber (Bogaert et al., 2000; Claus et al., 2015). Therefore, when the coronary artery is obstructing, myocardial ischemia gradually accumulates from the endocardium to the epicardium. Longitudinal fibers were the first to be affected, followed by oblique fibers and circumferential fibers. Therefore, the strain is reducing longitudinally, radially, and circumferentially (Neizel et al., 2009; Khan et al., 2015; Kihlberg et al., 2015). Regardless of the degree of transmurality, the longitudinal strain is first reduced, which further suggested that the circumferential strain reflects the degree of transmurality and is consistent with existing research (Mangion et al., 2017). This, in turn, suggests that the results obtained after comparing the AWMI group with the NAWMI group were caused by different transmurality.

Therefore, in the present study, we divided patients into two groups (transmurality ≤ 50% and transmurality > 50%; Ogawa et al., 2017). The obtained results revealed that GRS, GCS, and GLS of patients whose transmurality > 50% had a more remarkable decrease than patients with transmurality ≤ 50%. Thus, the greater degrees of transmurality implied the lower the strain value and the worse ability for myocardial deformation (Becker et al., 2009).

When patients were grouped by transmurality degree and then sub-grouped by the location of MI, the GCS and GLS are statistically reducing in the AWMI group compared to the NAWMI group. Besides, the IS was significantly different between the AWMI group and the NAWMI group. Previous studies have suggested that the infarct size was negatively correlated with the myocardial strain (larger size leads to lower strain) (Lund et al., 2007). Therefore, the decline of the GCS and GLS in the different groups could be related to IS, and covariance analysis was used to eliminate the effect of IS.

After infarct size was standardized in covariance analysis, there was no discrepancy in the GRS, GCS, and GLS between AWMI and NAWMI. The myocardial strain was related to infarct size rather than location. The inconsistency of the infarct size caused the difference between AWMI and NAWMI. After patients were grouped by transmurality degree and then sub-grouped by the location of MI, there was no discrepancy in the GRS, GCS, and GLS between the AWMI group and the NAWMI group. It demonstrated that the transmurality might not affect myocardial strain in the same level of IS between the AWMI group and the NAWMI group. However, the GRS and GCS were significantly different between the transmurality ≤ 50% group and the transmurality > 50% group, which demonstrated in the same level of IS, the different transmurality will lead to a different myocardial strain.

The present study demonstrated that the anterior wall leads to a worse prognosis because patients with AWMI usually have a larger infarct size, transmurality, and worse global left ventricular function, which is consistent with existing literature (Masci et al., 2011). This study showed that the AWMI has greater infarct size and transmurality, which revealed the fact that AWMI has a large number of myocardial necrosis. Therefore, it is necessary to focus on transmurality and infarct size in patients suffering from MI regardless of the MI location.

According to the guideline (O’Gara et al., 2013), the risk of different myocardial infarction location is different. However, the essence is that the infarct size and transmural degree are more in-depth from the present study. The infarct size is just the appearance. We should see through the appearance to perceive the essence, focus on the infarct size and transmurality, rather than just the location after infarction. Instead of risk assessment based on the location, we should classify the risk according to the infarct size and transmurality.

The present study has some limitations: (1) This study is a single-center study, and the multicentre study should further validate our conclusion; (2) Other studies indicate that the incidence rate of male and female myocardial infarction is about 1.68–1 (Mefford et al., 2020). About 10% of female patients were included, which did not conform to the actual proportion of male and female patients with myocardial infarction. However, in the selection of the control group, gender matching was carried out to eliminate the influence of gender on the results; (3) The acute phase and chronic phase were not grouped; (4) The approach of transmurality grouping adopted in this study was semi-quantitative; (5) the repeatability still needs to be improved by taking quantitative measures in the future.

## Data Availability Statement

The raw data supporting the conclusions of this manuscript will be made available by the authors, without undue reservation, to any qualified researcher.

## Ethics Statement

The studies involving human participants were reviewed and approved by the Ethics Committee and Institutional Review Board of Beijing Anzhen Hospital. The patients/participants provided their written informed consent to participate in this study.

## Author Contributions

SL wrote the manuscript. LZ and XM designed the study. SL, AL, and JT analyzed the data. SL, LZ, AL, JT, XM and LG reviewed the manuscript. All authors contributed to the article and approved the submitted version.

## Conflict of Interest

The authors declare that the research was conducted in the absence of any commercial or financial relationships that could be construed as a potential conflict of interest.
