# Supplementary material for: Comparison of Left Ventricular Global Strain in Anterior and Non-anterior Wall Myocardial Infarction With CMR Tissue Tracking
Source: Front Physiol. 2020 Dec 10;11:530108. doi: 10.3389/fphys.2020.530108 (PMC7758347; doi:10.3389/fphys.2020.530108)
Supplement: Supplementary file 1 [file Table_1.docx]

**Comparison of Left Ventricular Global Strain in Anterior and Non-Anterior Wall Myocardial Infarction with CMR Tissue Tracking**

**Authors’ names, academic degrees, and affiliations:**

Shuhao Li, Lei Zhao, Aijia Lu, Jie Tian, Lianggeng Gong, Xiaohai Ma

**Supplementary Table 1** Comparison of the MI location by transmurality degree

|  | transmural ≤50% | | | |  | transmural>50% | | | |
| --- | --- | --- | --- | --- | --- | --- | --- | --- | --- |
|  | AWMI | NAWI | F  value | *P*  value |  | AWMI | NAWMI | F  value | *P* value |
|  | （n=15） | （n=28） |  |  |  | （n=27） | （n=30） |  |  |
| GRS | 21±8 | 21±10 | 0.117 | 1.000 |  | 14±9 | 17±10 | 0.910 | 1.000 |
| GCS | -12±3 | -12±4 | 0.900 | 0.991 |  | -7±4 | -10±5 | 5.003 | 0.023 |
| GLS | -11±3 | -10±5 | 2.556 | 0.769 |  | -7±3 | -9±4 | 8.620 | 0.015 |
| IS | 17±7 | 10±7 | 0.008 | 0.006 |  | 25±9 | 17±8 | 0.028 | 0.001 |

**Supplementary Table 2** Comparison of AWMI and NAWMI by covariance analysis (%)

|  | AWMI  （n=42） | NAWMI  （n=58） | F value | *P* value |
| --- | --- | --- | --- | --- |
|  |  |  |  |  |
| GRS | 18±1 | 18±1 | 0.021 | 0.886 |
| GCS | -10±1 | -10±1 | 0.129 | 0.720 |
| GLS | -9±1 | -9±1 | 0.068 | 0.794 |

**Supplementary Table 3** Comparison by transmural degree by covariance analysis

|  | transmural ≤50% | | | | |  | | transmural>50% | | | | |  |
| --- | --- | --- | --- | --- | --- | --- | --- | --- | --- | --- | --- | --- | --- |
|  | | AWMI | NAWMI | F  value | *P*  value | |  | | AWMI | NAWMI | F  value | *P* value | |
|  | | （n=15） | （n=28） |  |  |  |  | | （n=27） | （n=30） |  |  |  |
| GRS | | 23±3 | 20±2 | 0.031 | 0.862 | |  | | 14±2 | 16±2 | 1.012 | 0.319 | |
| GCS | | -13±1 | -11±1 | 0.281 | 0.599 | |  | | -7±1 | -9±1 | 1.869 | 0.177 | |
| GLS | | -12±1 | -10±1 | 0.302 | 0.586 | |  | | -7±1 | -8±1 | 1.502 | 0.226 | |

**Supplementary Table 4** Comparison by transmurality degrees by covariance analysis (%)

|  | transmurality≤50%  （n=43） | transmurality>50%  （n=57） | F value | *P* value |
| --- | --- | --- | --- | --- |
| GRS | 20±1 | 16±1 | 4.290 | 0.041 |
| GCS | -11±1 | -9±1 | 6.387 | 0.013 |
| GLS | -10±1 | -8±1 | 3.857 | 0.052 |

**Supplementary Table 5** The Intra-class Correlation coefficient of strain and infarct size

|  |  | ICC | 95%CI | | P value | |
| --- | --- | --- | --- | --- | --- | --- |
| Intra-  Observer | GRS | 0.957 | 0.910 | 0.980 | <0.001 |  |
|  | GCS | 0.944 | 0.886 | 0.973 | <0.001 |  |
|  | GLS | 0.946 | 0.887 | 0.974 | <0.001 |  |
|  | IS | 0.911 | 0.814 | 0.958 | <0.001 |  |
| Inter-  Observer | GRS | 0.942 | 0.878 | 0.972 | <0.001 |  |
|  | GCS | 0.977 | 0.952 | 0.989 | <0.001 |  |
|  | GLS | 0.927 | 0.848 | 0.965 | <0.001 |  |
|  | IS | 0.927 | 0.848 | 0.965 | <0.001 |  |
